# Histological and Immunohistochemical Characterization of a Case of Endometriosis in a Guinea Pig* (Cavia tschudii)*

**DOI:** 10.1155/2017/4594510

**Published:** 2017-05-08

**Authors:** Alfonso Baldi, Andrea Lanza, Francesco Menicagli, Pietro G. Signorile, Enrico P. Spugnini

**Affiliations:** ^1^Department of Environmental, Biological and Pharmaceutical Sciences and Technologies, Second University of Naples, Caserta, Italy; ^2^Fondazione Italiana Endometriosi, Rome, Italy; ^3^Centro Veterinario Gianicolense, Rome, Italy; ^4^SAFU, Regina Elena Cancer Institute, Rome, Italy

## Abstract

Endometriosis is a chronic gynecological disease characterized by the ectopic proliferation of endometrial tissue outside of the uterine cavity. The pathogenesis of this disease is still obscure, and Sampson's theory of retrograde menstruation is still the most widely accepted explanation. Endometriosis in animals has been so far described not only in baboons and a rhesus macaque but also in dogs and horses that are nonmenstruating animals. In this article, we report the histological and immunohistochemical characterization of the first case of ovarian cystic endometriosis and adenomyosis in a guinea pig. The case presented supports the hypothesis that endometriosis is a disease not at all related to the phenomenon of retrograde menstruation but is a consequence of some alterations in the morphogenesis of the female genital system and therefore it could be found in any mammal. We suggest considering endometriosis among the other pathological phenotypes in animals displaying ovarian and uterine alterations and having a history of difficulties in conceiving.

## 1. Introduction

There is a clear scientific evidence that female reproductive efficiency has severely deteriorated over the past half century, especially in western countries such as USA [1, 2]. Among the causes of infertility, endometriosis is becoming a concerning increasing problem among the female population [3–5]. Endometriosis, a chronic gynecological disease characterized by the ectopic proliferation of endometrial tissue outside of the uterine cavity, is one of the most common causes of chronic pelvic pain and infertility, affecting up to 10% of women in the reproductive-age group and up to 30% in patients with infertility [6–8]. Adenomyosis is a particular form of endometriosis, where the ectopic endometrium is seen in the wall of the uterus. Several theories have tried to explain the causes of endometriosis and today Sampson's theory of retrograde menstruation is still the most widely accepted explanation. This theory postulates that endometriosis is caused by fragments of menstrual endometrium that refluxes through the fallopian tubes and then falls and implants within the peritoneal cavity [9]. However, although retrograde menstruation is widely reported among fertile women, endometriosis is only diagnosed in 10% of them [10, 11]. Despite the widespread acceptance of this theory, it is unable to justify the occurrence of this disease in extra-abdominal districts as well as the occurrence of male endometriosis [12–14]. Recently, a novel theory postulates the intervention of an altered exogenous estrogenic input during embryogenesis which would be responsible of the induction of reproductive disorders, including the development of endometriosis [15–17]. At the current stage of knowledge, endometriosis in animals has been, so far, reported only in dogs and horses that are nonmenstruating animals and, more recently, in baboons and a rhesus macaque [18–24]. Nevertheless, cystic ovaries reported in mammalians, including guinea pigs, horses, swine, rabbits, cattle, and aged rats, have been interpreted as to have been derived from corpora lutea, ovarian follicles, ovarian surface epithelium, remnants of rete ovarii, or mesonephric and paramesonephric ducts [25–27]. In this article, we report on a case of ovarian cystic endometriosis and adenomyosis in a guinea pig. The potential pathogenetic and clinical significance of endometriosis in animals and in particular of cystic ovarian endometriosis will be described.

## 2. Case Report

**Figure 1:**
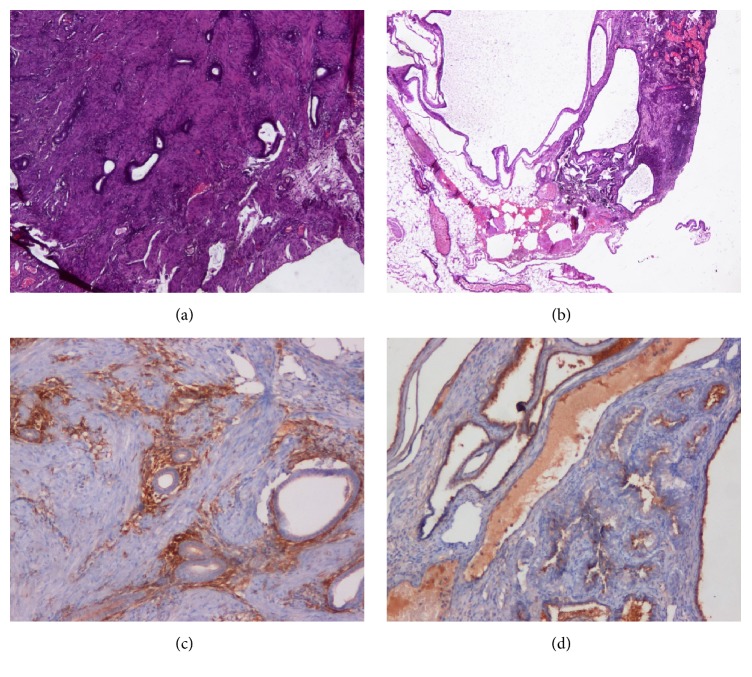
(a) Presence of ectopic endometrium in the uterine wall (H&E, original magnification ×10). (b) Cystic endometriosis present in the ovary (H&E, original magnification ×10). (c) The adenomyosis in the uterine wall is surrounded by a stromal reaction evidenced by the CD10 positivity (ABC, original magnification ×10). (d) The ectopic glandular epithelium of the cystic structure in the ovary expresses estrogen, as evidenced by immunohistochemistry with a monoclonal antibody specific for estrogen (ABC, original magnification ×10).

A privately owned two-year-old female guinea pig was referred for a repeated loss of material from the uterus and progressive weight loss. Anamnestically, the animal never conceived despite several attempts at mating. The day of the appointment, the pet expelled a large amount of hemorrhagic material. During the visit, the patient was quiet, depressed, and moderately responsive to stimulation. At physical exam, the patient showed tachypnea, vocalization upon manipulation of the abdomen that was tense, and dilated abdomen with a palpable mass. Ultrasonographic investigation evidenced a large well-defined 2 × 2 cm mass in the anatomic area of the uterus. The guinea pig underwent emergency surgery upon sedation with an association of butorphanol 0.7 mg/kg (Dolorex 10 mg/ml, MSD), medetomidine 0.07 mg/kg (Sedator 1,0 mg/ml, ATI), and ketamine 7 mg/kg (Imalgene 1000 100 mg/ml, MERIAL) administrated intramuscularly. Anesthesia was maintained with isoflurane 3% with a not cuffed endotracheal tube. The procedure was monitored with a multiparameter monitor, including ECG (II derivation) CO_2_, O_2_, and no invasive pressure and temperature. During the anesthesia, intravenous fluid (NaCl 0.9%) was administered at the rate of 5 ml/kg/h with infusion pump via intravenous catheter 24 G (Terumo). Surgical examination of the abdomen evidenced an enlarged and congested ovary and uterus and signs of peritonitis, including intra-abdominal fluid. The two organs were excised and submitted for histopathology. The patient was discharged on antibiotic (enrofloxacin 5 mg/kg bid/po Baytril flavour sosp os 25 mg/ml Bayer) and nonsteroidal anti-inflammatory drugs (meloxicam 0.3 mg/kg/sid/po Metacam sosp os 1,5 mg/ml flac 10 ml Boheringer) and ranitidine (3 mg/kg/bid/po Zantadine sol os 30 mg/ml Ceva) [28]. Histopathologic analysis revealed characteristic features of endometriosis both in the uterus and in the ovary. In detail, adenomyosis was described in the uterus because of the presence of ectopic glandular tissue in the muscular wall of the uterus (Figure 1(a)). On the other hand, at the level of the ovary, cystic enlargements filled with fluid were seen macroscopically; histologically, these cystic structures presented the classic glandular epithelium of the endometrium with one layer of cuboidal or tall cells, thus representing classic cystic endometriosis of the ovary (Figure 1(b)). Immunohistochemical staining was performed, by using the ABC method and diaminobenzidine, by means of specific antibodies for estrogen and CD10 in order to confirm the ability of the glandular epithelium to secrete estrogen and the presence of a stromal reaction surrounding the ectopic endometrial tissue (Figures 1(c) and 1(d)) [15, 16]. The histopathological and immunohistochemical features described are strongly suggestive of endometriosis.

The guinea pig recovered from the surgery and was rechecked on a monthly basis. The patient died of unrelated causes three months later. A necropsy was performed at that time and did not show any sign of endometriosis.

## 3. Discussion

Cystic lesions of the ovary are commonly seen in mammalians and have been described as lutein cysts and ovarian epithelial cysts or Graafian follicle cysts [25]. On the other hand, there are scant reports of ectopic endometrial tissue in domestic animals. Some reports describe the immunohistochemical investigation of mesonephric remnants that behave as ectopic endometrium in dogs [18, 19]. In detail, the mesonephric remnants have been described in three bitches, and these findings were associated with abnormal reproductive behavior [19]. Several studies and case reports describe those remnants as “Gartner cysts” or “Gartner duct cysts” surrounded by inner circular and outer longitudinal layers of smooth muscle cells [19]. In this article, we report on a case of endometriosis, involving both the uterus and the ovary, in a guinea pig. To the best of our knowledge, this is the first case of endometriosis described in guinea pig. To date, the retrograde menstruation theory is still the most widely recognized explanation for the pathogenesis of endometriosis [9]. Therefore, endometriosis has been considered as a disease not present in nonmenstruating animals. Lately, scientists have suggested some different modalities for its pathogenesis, with retrograde menstruation, coelomic metaplasia, and endometrial stem cells being the most well-acknowledged hypotheses [17].

Recently, our research group has shown the presence of an ectopic endometrium with a molecular and histological phenotype identical to that of the eutopic endometrium in a substantial number of human female fetuses analyzed by postmortem dissection [15, 29, 30]. These findings suggest that molecular events acting in a critical window of time during embryogenesis could cause a perturbation of the fine-tuning mechanisms responsible for the correct development of the female genital system. This, in turn, would consent the displacement of endometrial tissue during the earlier stages of organogenesis. It is possible that an anomalous estrogenic input, acting on a favorable genetic background, could represent the basis of this phenomenon [16, 31].

The case presented, together with the few data presented in the current literature about endometriosis in mammalians, supports the hypothesis that endometriosis is a disease related to some alterations in the morphogenesis of the female genital system and therefore it could be found in several mammals. It is possible that (with endometriosis being a disease that cannot be clearly diagnosed with serological and instrumental analysis) its presence and incidence in animals are largely underestimated. Nevertheless, we suggest considering endometriosis among the other pathological phenotypes in animals displaying ovarian and uterine alterations and having a history of difficulties in conceiving.
